# Supplementary material for: Progesterone Receptor Expression Level Predicts Prognosis of Estrogen Receptor-Positive/HER2-Negative Young Breast Cancer: A Single-Center Prospective Cohort Study
Source: Cancers (Basel). 2023 Jun 30;15(13):3435. doi: 10.3390/cancers15133435 (PMC10341192; doi:10.3390/cancers15133435)
Supplement: Supplementary file 1 [file cancers-15-03435-s001.zip › cancers-2407243-supplementary.pdf]

Table S1. Pathologic stage according to neoadjuvant chemotherapy.

p stage (without neoadjuvant chemotherapy)				
	**Strong-PR (*n* = 318, 89.6%)**	**Low-PR (*n* = 13, 3.7%)**	**PR-negative (n=24, 6.8%)**	***p* value**
**pT**				0.4250
T1	180 (56.6)	6 (46.2)	14 (58.3)	
T2	119 (37.4)	5 (38.5)	10 (41.7)	
T3	19 (6.0)	2 (15.4)	0 (0.0)	
**pN**				0.0420
N0	200 (62.9)	7 (53.8)	19 (79.2)	
N1	86 (27.0)	3 (23.1)	4 (16.7)	
N2	25 (7.9)	0 (0.0)	1 (4.2)	
N3	7 (2.2)	3 (23.1)	0 (0.0)	
**pStage**				0.5700
I	147 (46.2)	5 (38.5)	12 (50.0)	
II	132 (41.5)	5 (38.5)	11 (45.8)	
III	39 (12.3)	3 (23.1)	1 (4.2)	

yp stage (with neoadjuvant chemotherapy)				
	**Strong-PR (n=68, 66.0%)**	**Low-PR (n=13, 12.6%)**	**PR-negative (n=22, 21.4%)**	***p* value**
**ypT**				0.6670
pCR	4 (5.9)	2 (15.4)	3 (13.6)	
T1	30 (44.1)	6 (46.2)	12 (54.5)	
T2	23 (33.8)	4 (30.8)	5 (22.7)	
T3	11 (16.2)	1 (7.7)	2 (9.1)	
**ypN**				0.3820
N0	19 (27.9)	5 (38.5)	12 (54.5)	
N1	27 (39.7)	5 (38.5)	7 (31.8)	
N2	15 (22.1)	3 (23.1)	2 (9.1)	
N3	7 (10.3)	0 (0.0)	1 (4.5)	
**ypStage**				0.2820
pCR	4 (5.9)	2 (15.4)	3 (13.6)	
I	12 (17.6)	2 (15.4)	8 (36.4)	
II	27 (39.7)	5 (38.5)	7 (31.8)	
III	25 (36.8)	4 (30.8)	4 (18.2)	
